# Supplementary figures and images for: Myc-regulated miRNAs modulate p53 expression and impact animal survival under nutrient deprivation
Source: PLoS Genet. 2023 Aug 28;19(8):e1010721. doi: 10.1371/journal.pgen.1010721 (PMC10491395; doi:10.1371/journal.pgen.1010721)

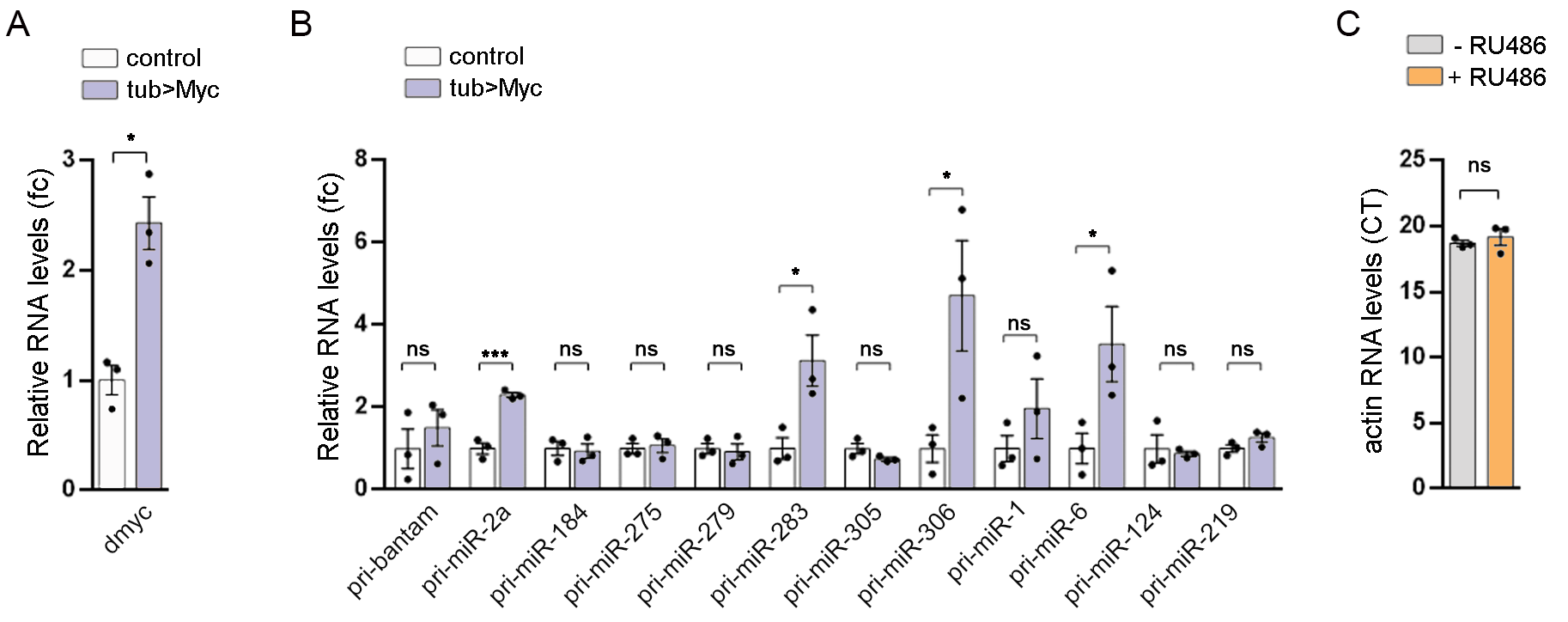

Supplement: S1 Fig — (A) qRT-PCR showing myc mRNA levels in control (w1118) and tub>dMyc larvae. (B) qRT-PCR showing pri-miR expression in control (w1118) and tub>dMyc larvae. Results are expressed as fold induction with respect to control animals. (C) qRT-PCR showing that actin mRNA levels remained unaffected in GSG162>mycRNAi larvae with either RU486 or vehicle treatment. The results are presented as CT (cycle threshold) values for each condition. Mean ± SEM. Unpaired two-tailed t-tests: * p<0.05; ** p<0.01; ***p<0.001; ns: not significant. (TIF) [file pgen.1010721.s001.tif]

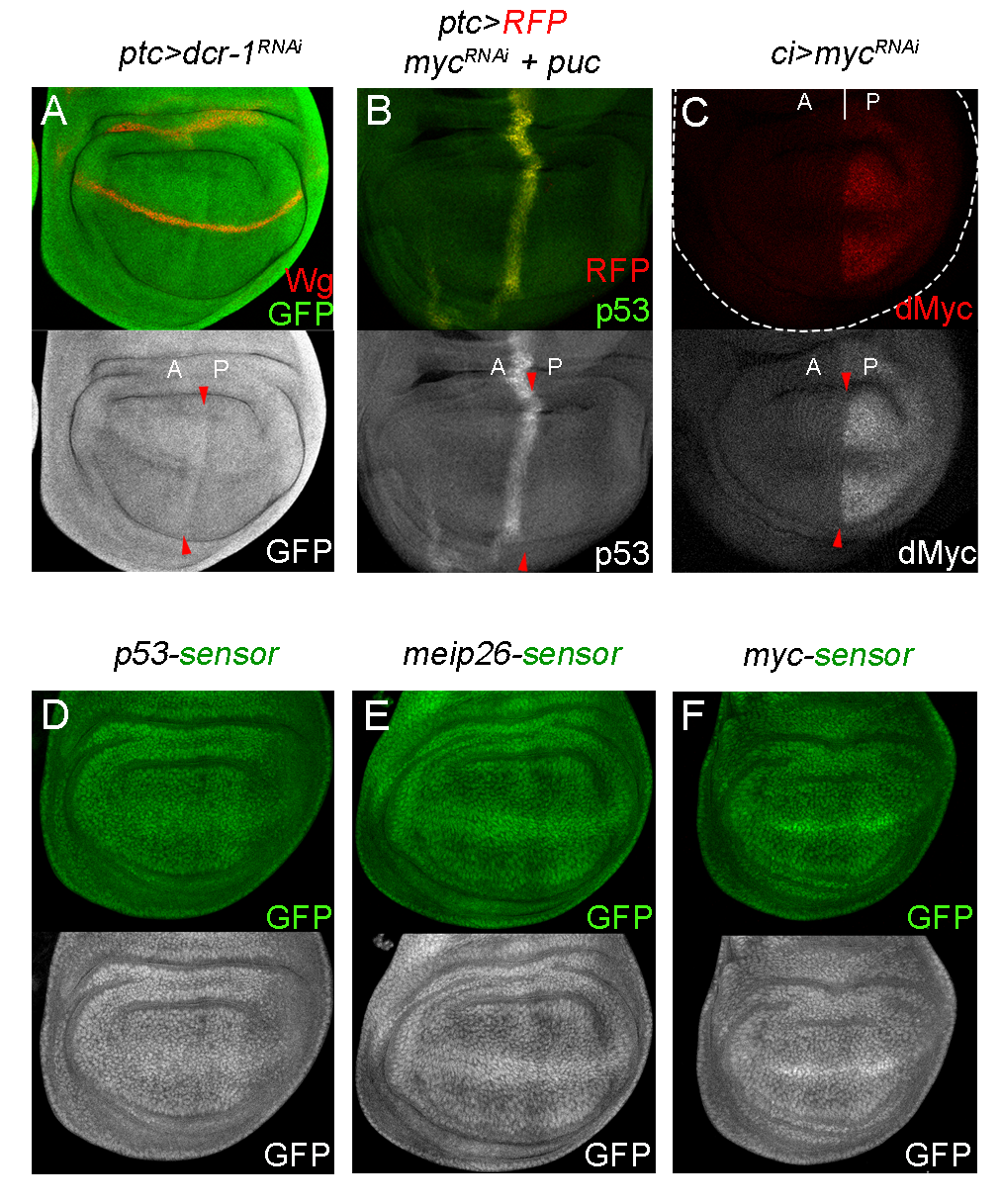

Supplement: S3 Fig — (A) Wing discs carrying the p53-sensor and expressing dcr-1RNAi under the control of the ptc-Gal4 driver (marked by the expression of RFP, in red) stained to visualize GFP (in green or white). (B) Wing discs expressing mycRNAi under the control of the ptc-Gal4 driver (marked by the expression of RFP, in red) and stained to visualize Dmp53 protein expression (in green or white). (C) Wing discs expressing mycRNAi under the control of the ci-Gal4 driver and stained to visualize dMyc protein expression (in red or white). (D-F) Wing discs carrying the indicated miR-sensors and stained to visualize GFP (in green or white). Red arrowheads depict the anterior-posterior (A-P) boundary. A: anterior; P: posterior. (TIF) [file pgen.1010721.s003.tif]

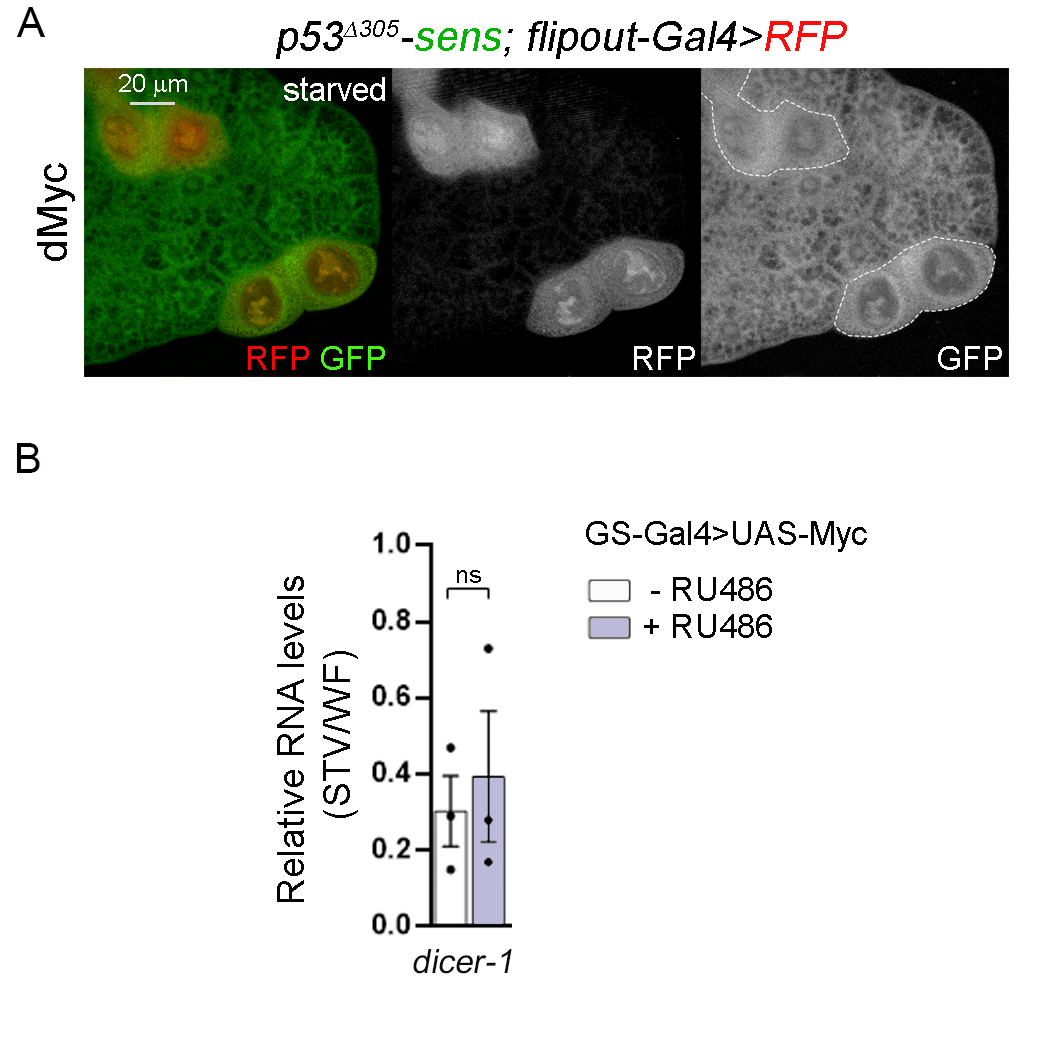

Supplement: S4 Fig — (A) Fat body cells labeled to visualize p53Δ305-sensor (in green or white) from starved larvae expressing dMyc (marked by the expression of RFP, in red or white). Scale bars, 20 μm. (B) qRT-PCR showing dcr1 transcript levels in the FB of larvae from the indicated genotypes subjected to well fed (WF) or starved (STV) conditions. Results are expressed as fold induction with respect to control animals. Mean ± SEM. Unpaired two-tailed t-tests: ns: not significant. (TIF) [file pgen.1010721.s004.tif]

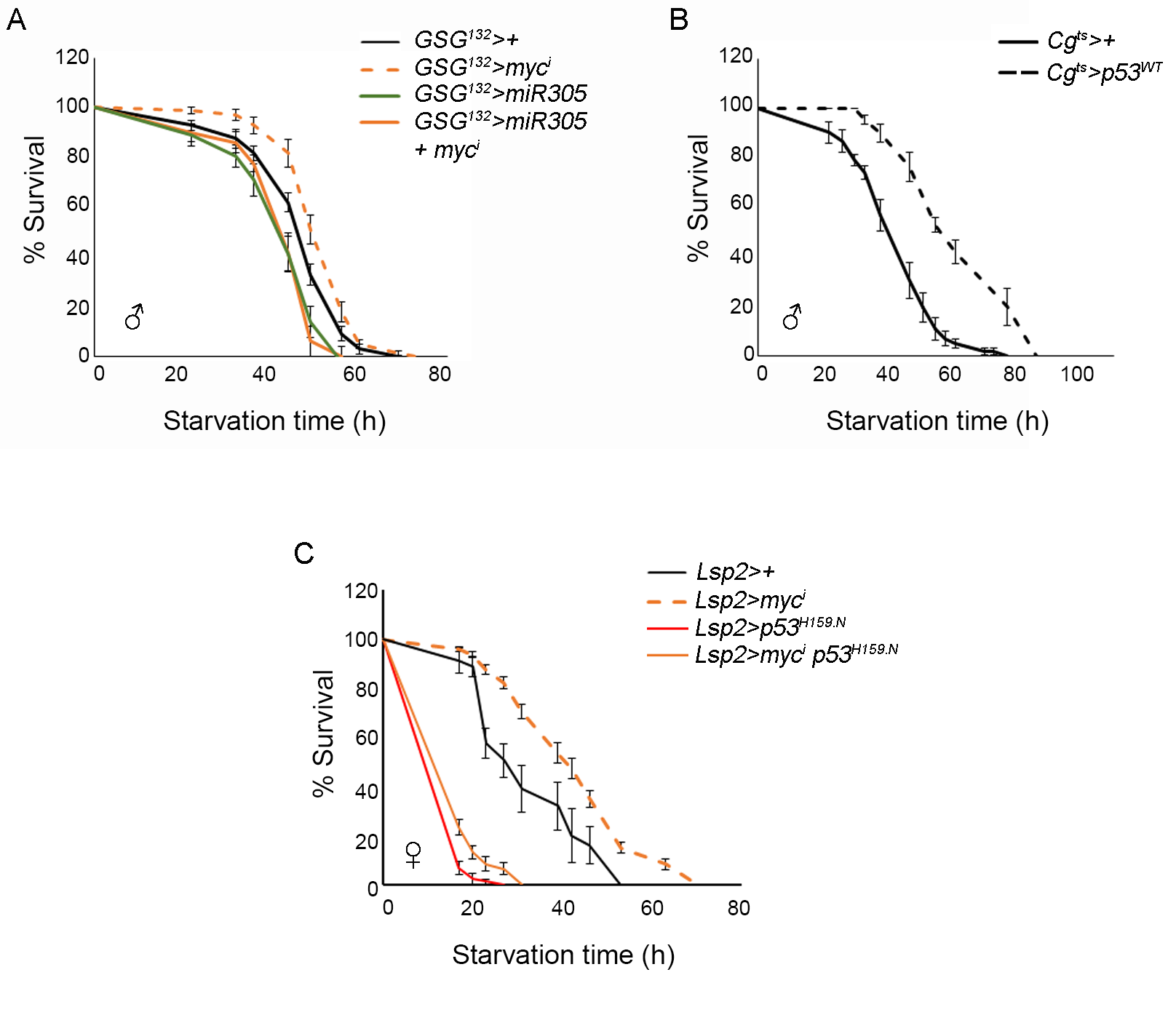

Supplement: S5 Fig — (A-C) Survival rates to nutrient deprivation of adult flies of the indicated genotypes compared to control flies subjected to the same procedure. (A) The GSG132-Gal4 line was utilized to express dMyc or miR-305 specifically in the adult fat body. Newly eclosed adults of each genotype were transferred to food supplemented with 50 μg/ml of RU486 (Sigma). After 5 to 7 days, flies were transferred to vials containing 2% agar in PBS along with 50 μg/ml of RU486. (B) The Cg-Gal4, tub-Gal80ts line was used to express Dmp53 in the adult fat body. Newly eclosed adults of each genotype, grown at 18°C, were switched to 29°C. After 5 to 7 days at 29°C, flies were transferred to vials containing 2% agar in PBS. See S2 Table for n, p-value, median, and maximum survival values. Error bars represent SEM. (TIF) [file pgen.1010721.s005.tif]
