# Supplementary material for: Myc-regulated miRNAs modulate p53 expression and impact animal survival under nutrient deprivation
Source: PLoS Genet. 2023 Aug 28;19(8):e1010721. doi: 10.1371/journal.pgen.1010721 (PMC10491395; doi:10.1371/journal.pgen.1010721)
Supplement: Supplementary file 2 [file pgen.1010721.s002.docx]

A

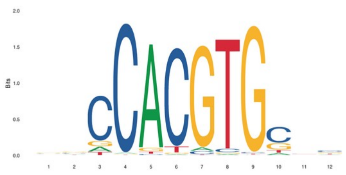

Myc HS
MA0147.3

B

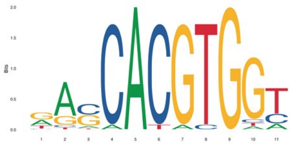

Myc:MAX HS
MA0059.1

C

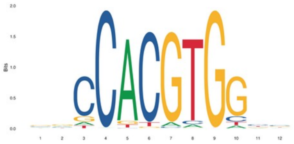

MycN HS
MA0104.4

D

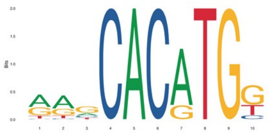

Imperfect E-box
(Max like)

E

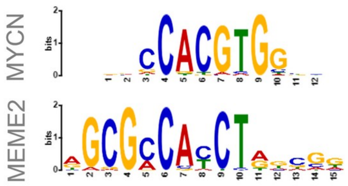

MEME2 (Highly enriched
Myc E-box motif like)
