# Supplementary material for: Myc-regulated miRNAs modulate p53 expression and impact animal survival under nutrient deprivation
Source: PLoS Genet. 2023 Aug 28;19(8):e1010721. doi: 10.1371/journal.pgen.1010721 (PMC10491395; doi:10.1371/journal.pgen.1010721)
Supplement: Attachment — Submitted filename: renamed_e1ae3.pdf [file pgen.1010721.s009.pdf]

Revision report of Gervé et al. Myc-regulated miRNAs modulate p53 expression and impact animal survival under nutrient deprivation

The authors show that dMyc regulates a large share of miRNA genes as well as the miRNA machinery in the fly larva. They then use a miRNA sensor to show the regulation of dp53 by dMyc and miR305 in vivo, in wing disc and in fat body. Finally, the authors demonstrate a physiological relevance for the dMyc-miR305-dp53 pathway under nutrient deprivation.

Overall, the manuscript is well written, the methods and the data are clearly presented and for the most part the data is supporting the conclusions. My main concern is that it is unclear to what extent this study expands the already known mechanism published by the corresponding author previously (https://doi.org/10.1016/j.celrep.2014.06.020). In Barrio et al. (Cell Reports 2014) it was shown that TOR-miR305-dp53 pathway in the fat body promote survival under nutrient stress. While the finding that Myc is a major regulator of miRNAs is potentially interesting, it is also well known that Myc is acting downstream of TOR signaling. Hence, the finding that Myc is a mediator in their previously published mechanism is, to some extent, expected.

Specific comments figure by figure:

Fig 1.
Please indicate the applied statistical test used in the Figure legend (applies to all figure legends).

Since dMyc is a major regulator of cellular growth, its downregulation is expected to reduce the level of overall transcription as well. To make the results more convincing, could the authors provide the data of their reference gene actin, to show its expression has remained constant in their experiments. This data can be provided in the supplement.

The authors conclude "Together, these findings indicate that dMyc regulates the expression of a large number of miRNAs in Drosophila", yet the number of affected pri-miRNAs between dMyc LOF and GOF are quite different (10 in LOF vs. 3 in GOF). Could the authors clarify in the text why such difference exists?

Figure 1 title states that "Myc induces widespread expression of miRNA genes in Drosophila". Could the authors rephrase the title since the word "induces" refers to Myc upregulating the miRNA levels which is not shown here.

Fig 2.

The conclusions are justified by the provided data.

Fig 3.

To make the regulatory function of dMyc to the elements of the miRNA machinery more clear, could the authors also show the dMyc GOF data for the ago1, dcr-1 and drosha expression?

Fig 4.

It is not completely clear why the results from the meip26-sensor and myc-sensor are shown. Could the authors clarify this in the text?

To make the results more convincing, a quantification of the GFP signal along the wing disc A-P axis is required.

In (A), the RFP signal is missing from the figure.

Fig 5.

In S1 pri-mir305 was not affected by dMyc overexpression, yet in Fig5 it is affected by dMyc overexpression. The reason might be that the measurements are from different tissues. Could the authors clarify this in the text?

Could the authors change ‘well fed’ into ‘fed’ and STV into starv? This would make the interpretation of the figure easier for the reader.

In panel C, the authors show that dcr-1 and drosha levels were not rescued by the expression of dMyc in starved versus fed larvae. Based on this a conclusion of an indirect regulation was made. However, in 3B, by ChIP-qPCR, the authors show the direct binding of dMyc into the drosha promoter. Could the authors clarify this discrepancy?

Could the authors clarify the details of the clone technique used in Fig5 E & F in the figure legend? This would make it easier for the reader to understand the nature of the experiment. Please also clarify the used ‘AFGal4’ driver.

Fig 6.

Is the dMyc and miR305 knockdowns present throughout the fly development, and if yes, what are the consequences of dMyc and mir305 manipulation during development to the adult flies? It would be important to understand if the adult starvation sensitivity/insensitivity is due to developmental defects or specific to the adult stage.

The results from overexpressing p53 in the fat body are not shown. According to the model, overexpressing p53 in the fat body should increase starvation sensitivity. To make the conclusions solid, this result should be provided. Alternatively, a reason for not conducting this experiment should be provided.

In panel E, the Lsp2>+ is not shown. Also the data is only shown for male flies. To make the conclusion of the myc-p53 interaction in starvation sensitivity convincing, the authors should provide the Lsp2>+ control, and the female data (even if negative). Or at least provide a reason why the female data was omitted.

Please indicate the fly gender also in the figure panels.

In line 182 is mentioned Fig 6F, but this panel is not included in figure 6.

A model figure explaining the dMyc-mir305-p53 pathway would be helpful for the reader.
