# Supplementary material for: Myc-regulated miRNAs modulate p53 expression and impact animal survival under nutrient deprivation
Source: PLoS Genet. 2023 Aug 28;19(8):e1010721. doi: 10.1371/journal.pgen.1010721 (PMC10491395; doi:10.1371/journal.pgen.1010721)
Supplement: Attachment — Submitted filename: Response to Reviewers .docx [file pgen.1010721.s010.docx]

**Point-by-point response to the reviewers' comments on manuscript PGENETICS-D-23-00345**

**Reviewer #1:** In this paper, Gerve et al describe a role for Myc as a regulator of miRNA expression and processing. They re-analyzed published ChIP seq data sets and found that Myc can directly bind to miRNA promoter regions and an also control the expression of many miRNAs. They also show that one Myc-regulated miRNA (miR305) is important in mediating the effects of adipose Myc on starvation survival.

Overall, this was a very good paper. The experiments were all well-performed and the interpretations and conclusions were supported by the findings. This study extends our appreciation of Myc target genes and how Myc controls gene expression. In this context, the work will be of interest to a broad array of researchers interested in Myc function in different systems (not just Drosophila).

We appreciate the reviewer's positive comments on our work and all the suggestions. As detailed below, we have included control images for each miR-sensor and quantified GFP intensity levels. We have also added additional experiments to reinforce our conclusion that Myc is not involved in controlling Dcr-1 levels in the FB of starved animals.

These are some minor issues that the authors may choose to address before publication.

Fig 4: The effects of Myc overexpression/knockdown on the Myc sensor seem quite weak. In fig 4D also looks like the down regulation of the myc-sensor is non-autonomous – the stripe of decreased expression of the sensor seems wider than the dpp>RFP expression domain. Is this case? If so, it might be worth commenting on. Or is there a lower expression level in this domain in the absence of myc expression. For this figure, it would be good to add a control image for each sensor showing the expression pattern of the sensor in the absence of Myc expression.

We greatly appreciate the reviewer for pointing out this observation. We acknowledge that the discrepancy between the Red and Green channels may be confusing. However, it is important to clarify that this disparity does not arise from a non-cell autonomous role of Myc in controlling miR-sensor levels. Instead, it stems from the merge image representation. In this specific focal plane, the signal from RFP is not as good as the GFP signal. If we adjust the image saturation, it becomes evident that the expression domain is indeed the same (see below).

On the other hand, as recommended by the reviewer, we have incorporated control images displaying the expression pattern of each miR-sensor in Figure S4. Notably, no discernible differences in GFP expression along the anterior-posterior (A-P) axis were observed. Additionally, we have quantified the intensity levels of GFP for each miR-sensor and genetic condition analyzed in Figure 4. These results have been included as Figure 4D, and text has been modified accordingly. We are confident that these new data effectively demonstrate the impact of Myc on the regulation of miR-sensor levels.

Fig 5. In Fig 5C, the authors use the tub>Myc system to see if Myc overexpression can reverse the starvation-induced decrease in dcr1. They saw ~3fold upregulation of Myc mRNA but no reversal of the starvation mediated decreased. However, starvation can posttranscriptionally suppress Myc levels and so I wonder whether there is sufficient upregulate of Myc protein levels with the tub>Myc system. Would it be worth trying a stronger Myc expression, maybe using heatshock induction with the hsflp-out system (Fig 5E, F).

As recommended, we have used the geneswitch-Gal4/UAS system to overexpress Myc (similar to our approach for Myc LOF experiments shown in Fig 1, 3, 5). Interestingly, the reduced levels of Dcr-1 observed in the fat body of starved control larvae (GSG162>Myc without RU486) were not reverted by Myc overexpression (GSG162>Myc with RU486). We have included this data as Figure S5B, and text has been revised as follows:

Line 173: “Similar results were obtained by overexpressing dMyc with the GSG162-Gal4 driver in the presence of RU486 (Fig. S5).”

Reviewer #2: Revision report of Gervé et al. Myc-regulated miRNAs modulate p53 expression and impact animal survival under nutrient deprivation

The authors show that dMyc regulates a large share of miRNA genes as well as the miRNA machinery in the fly larva. They then use a miRNA sensor to show the regulation of dp53 by dMyc and miR305 in vivo, in wing disc and in fat body. Finally, the authors demonstrate a physiological relevance for the dMyc-miR305-dp53 pathway under nutrient deprivation.
Overall, the manuscript is well written, the methods and the data are clearly presented and for the most part the data is supporting the conclusions. My main concern is that it is unclear to what extent this study expands the already known mechanism published by the corresponding author previously (<https://doi.org/10.1016/j.celrep.2014.06.020>). In Barrio et al. (Cell Reports 2014) it was shown that TOR-miR305-dp53 pathway in the fat body promote survival under nutrient stress. While the finding that Myc is a major regulator of miRNAs is potentially interesting, it is also well known that Myc is acting downstream of TOR signaling. Hence, the finding that Myc is a mediator in their previously published mechanism is, to some extent, expected.

We appreciate the reviewer's positive comments on our work and all the suggestions that have greatly improved the quality of the manuscript. As detailed below, we have added additional experiments to reinforce our conclusions and have discussed important issues accordingly. We have also thoroughly revised the manuscript to clearly state the novelty of the results.

Regarding the reviewer's concern about novelty, we would like to emphasize that our previous work established the significant role of miR-305 in regulating Dmp53 according to nutrient availability. In this study, we have taken a step further by investigating the involvement of dMyc in miRNA gene expression and processing. Our findings reveal a direct regulatory role of dMyc on numerous miRNAs, with a particular focus on miR-305 expression in the fat body. Importantly, we observed that reduced dMyc levels during nutrient deprivation lead to the downregulation of pri-miR-305, which facilitates Dmp53 activation, ensuring energy homeostasis and survival under nutrient stress. Furthermore, we have recently uncovered FOXO-mediated repression of Dicer-1 in response to nutrient deprivation, highlighting the intricate interplay of different transcription factors in controlling miRNA biogenesis. Overall, our study provides new insights into the dynamic mechanisms governing miRNA-mediated responses to changing nutrient conditions and their crucial role in organismal survival.

Specific comments figure by figure:

Fig 1.

Please indicate the applied statistical test used in the Figure legend (applies to all figure legends).
Since dMyc is a major regulator of cellular growth, its downregulation is expected to reduce the level of overall transcription as well. To make the results more convincing, could the authors provide the data of their reference gene actin, to show its expression has remained constant in their experiments. This data can be provided in the supplement.
The authors conclude “Together, these findings indicate that dMyc regulates the expression of a large number of miRNAs in Drosophila”, yet the number of affected pri-miRNAs between dMyc LOF and GOF are quite different (10 in LOF vs. 3 in GOF). Could the authors clarify in the text why such difference exists?

Figure 1 title states that “Myc induces widespread expression of miRNA genes in Drosophila”. Could the authors rephrase the title since the word “induces” refers to Myc upregulating the miRNA levels which is not shown here.

In response to the suggestions, we have made several improvements to the manuscript. Firstly, we have included the statistical tests used in the Figure legends. Additionally, we have presented the levels of actin transcripts in dMyc depleted animals in Figure S1C. Lastly, we have modified the title to "*Myc-mediated regulation of miRNA gene expression in Drosophila*".

Regarding the number of affected pri-miRNAs between dMyc loss-of-function (LOF) and gain-of-function (GOF) experiments, we have included new data on Myc overexpression experiments to match the same number of pri-miRNAs analyzed in the Myc LOF experiments (as suggested by Reviewer 3). However, there is still a discrepancy in the number of affected pri-miRNAs between dMyc LOF and GOF experiments, with 9 in LOF compared to 4 in GOF.

It is possible to speculate that the lack of significant effects on miRNA transcription in the presence of additional dMyc could be due to the saturation of dMyc occupancy on the promoter regions of most miRNA genes. This suggests that the binding sites for dMyc on these promoters might already be fully occupied, resulting in limited impact on their transcription. This aligns with the fact that qPCR experiments were conducted using samples from actively growing L3 larvae, where Myc expression levels are known to be high.

This new data has been added in Fig. S1 and text modified as follows:

Line 97: “*We then evaluated the effect of overexpressing dMyc on pri-miRNA expression by using a transgenic line expressing dMyc under the control of a tubulin promoter (tub>dMyc; [18]) which resulted in a two to three-fold increase in dmyc transcript levels (Fig. S1). In this case, we observed the upregulation of pri-miR-283, pri-miR-306, pri-miR-6, and pri-miR-2a, while no significant differences were detected for other pri-miRNAs (Fig. S1). The absence of significant effects on miRNA transcription upon dMyc overexpression may be attributed to the saturation of dMyc occupancy on the promoter regions of miRNA genes. Collectively, these findings provide compelling evidence for the regulatory role of dMyc in controlling the expression of a substantial number of miRNAs in Drosophila.”*

Fig 2.

The conclusions are justified by the provided data.

Fig 3.

To make the regulatory function of dMyc to the elements of the miRNA machinery more clear, could the authors also show the dMyc GOF data for the ago1, dcr-1 and drosha expression?

As suggested, we have incorporated the measurement of *ago-1, drosha, and dicer-1* mRNA levels in dMyc gain-of-function (GOF) experiments. This data has been included in Fig. 3B and explained in text as follows:

Line 131: “*Interestingly, the transcript levels of dcr-1, drosha and ago1 were strongly reduced in larvae expressing mycRNAi (Fig. 3A), whereas they were upregulated in tub>dMyc animals (Fig. 3B).”*

Figure Legend: “*(A-B) qRT-PCR showing dcr1, drosha and ago1 mRNA levels in GSG162>mycRNAi larvae treated with RU486 or vehicle (A), as well as in tub>dMyc larvae (B). Results are expressed as fold induction relative to control animals. Three independent replicates were carried out for each sample.*”

Fig 4.

It is not completely clear why the results from the meip26-sensor and myc-sensor are shown. Could the authors clarify this in the text?

To make the results more convincing, a quantification of the GFP signal along the wing disc A-P axis is required.

In (A), the RFP signal is missing from the figure.

We appreciate the reviewer for pointing out this observation. Initially, our intention was to investigate the potential regulatory role of Myc in miRNA biogenesis beyond the miRNAs targeting p53. In response to this, we decided to expand our analysis by including additional miRNA sensors.

We have rephrase text in order to clarify this point, as follows:

Line xx: “We then extended our analysis to include alternative miR-GFP sensors based on Mei-p26 or dMyc 3'UTRs, which have previously been demonstrated to be regulated by miRNAs in the Drosophila wing imaginal disc [29,30]. Intriguingly, dMyc-depletion increased the expression of these miR-GFP sensors (Fig. 4D,E,G) and dMyc overexpression resulted in a strong reduction of GFP levels (Fig. 4D,F,H) compared with control discs showing ubiquitous GFP expression (Fig. S4). These findings indicate that the regulatory influence of dMyc extends beyond miRNAs targeting p53. Moreover, our results indicate that dMyc plays a crucial role in regulating the miRNA machinery in the *Drosophila* wing primordium, influencing either miRNA gene expression or processing.”

In addition, as recommended by the reviewer, we have quantified the intensity levels of GFP for each miR-sensor and genetic condition analyzed in Figure 4. These results have been included as Figure 4D, and text has been modified accordingly. We are confident that these new data effectively demonstrate the impact of Myc on the regulation of miR-sensor levels.

Figure 4A has been replaced to include RFP signal.

Fig 5.

In S1 pri-mir305 was not affected by dMyc overexpression, yet in Fig5 it is affected by dMyc overexpression. The reason might be that the measurements are from different tissues. Could the authors clarify this in the text?

In dMyc loss-of-function (LOF) experiments, we observed a decrease in pri-miR-305 expression (Figures 1B and 5B). However, in Myc gain-of-function (GOF) experiments, pri-miR-305 was not upregulated (Figures S1). The absence of upregulation in Myc GOF experiments may be attributed to the saturation of Myc occupancy on the promoter region of pri-miR-305, as discussed before. The results presented in Figure 5D demonstrate that Myc overexpression can rescue the reduced levels of pri-miR-305 observed under starvation conditions. However, it does not have an effect on miR-305 levels in fed conditions, which is consistent with the data shown in Figure S1. We hope that this explanation helps clarify the reviewer's concern.

Could the authors change ‘well fed’ into ‘fed’ and STV into starv? This would make the interpretation of the figure easier for the reader.

We have change ‘well fed’ into ‘fed’ and ‘STV into starved

In panel C, the authors show that dcr-1 and drosha levels were not rescued by the expression of dMyc in starved versus fed larvae. Based on this a conclusion of an indirect regulation was made. However, in 3B, by ChIP-qPCR, the authors show the direct binding of dMyc into the drosha promoter. Could the authors clarify this discrepancy?

We appreciate the reviewer for bringing up this point. Our findings demonstrate that despite being a direct target of Myc, the overexpression of dMyc does not restore the reduced levels of Drosha mRNA observed in the fat body during starvation (Fig. 5C). This suggests the involvement of additional transcriptional or post-transcriptional mechanisms in the regulation of Drosha expression.

We have discussed this point in text as follows:

Line 180: *“Together, these findings suggest that the transcription of miR-305 is directly regulated by dMyc, while the expression of Dcr-1 and Drosha in the fat body under nutrient deprivation involves a dMyc-independent mechanism. Interestingly, a recent study has reported that the transcription factor FOXO represses Dcr-1 expression levels under nutrient deprivation, indicating the involvement of alternative regulatory pathways [34]. Further investigation is required to determine whether Drosha is also negatively regulated under nutrient stress.”*

Could the authors clarify the details of the clone technique used in Fig5 E & F in the figure legend? This would make it easier for the reader to understand the nature of the experiment. Please also clarify the used ‘AFGal4’ driver.

As suggested by the reviewer, we have replaced AFGal4 with flipout-Gal4 in Figure 5 and provided a more detailed description of the mosaic analysis shown in Fig. 5E-F:

Figure legend: “*(E-F) The flipout-Gal4 line was used to overexpress dMyc in single fat body cells, allowing us to investigate the influence of dMyc expression on p53-sensor levels. Notably, expression of dMyc in single fat body cells (marked by RFP, in red or white) led to a decrease in p53-sensor levels of both fed and starved animals (E; in green or white), while having no effect on the expression of the p53Δ305-sensor (F; in green or white).*”

Fig 6.

Is the dMyc and miR305 knockdowns present throughout the fly development, and if yes, what are the consequences of dMyc and mir305 manipulation during development to the adult flies? It would be important to understand if the adult starvation sensitivity/insensitivity is due to developmental defects or specific to the adult stage.

In order to address reviewer concern, we have performed new experiments showing that conditional expression of myc^RNAi^ in the FB of adult flies by using the geneswitch^132^-Gal4 line increased survival rates upon STV (new Fig. S6A). Conversely, expression of miR305 specifically in the adult FB (GSG^132^>UAS-miR-305) showed reduced survival to nutrient deprivation (new Fig. S6A). These results suggest that the starvation sensitivity/insensitivity is more likely due to an effect of manipulating the levels of dMyc or miR-305 in the adult stage.

In the case of miR-305 depletion (Fig. 6C-D), expression of miR-305-sponge in the FB by using Lsp2-Gal4 driver (Lsp2>miR-305^sp^) showed similar survival rates than control (Lsp2>+) flies, suggesting there are no developmental defects associated to FB-miR305 depletion impacting on animal survival.

This new data has been included as Figure S6 and commented in text as follows:

Line 201: “*Similar results were obtained when modulating dMyc or miR-305 levels specifically in the adult FB using the GSG132-Gal4 driver in the presence of RU486 (Fig. S6).*”

Fig. S6 legend: “*(A-B) Survival rates to nutrient deprivation of adult ﬂies (males) of the indicated genotypes compared to control flies subjected to the same procedure. See Table S2 for n, p-value, median, and maximum survival values. Error bars represent SEM.*”

*The weaker phenotype observed in GSG132>UAS-miR-305 (Fig. S6) compared to Lsp2>UAS-miR-305 (Fig. 6A) may be due to the differing strengths of the Gal4 drivers, with Lsp2-Gal4 being notably stronger than GSG132-Gal4 in our RU486 induction setup. Alternatively, these differences could be attributed to potential unintended effects resulting from the overexpression of miR-305 during development.

The results from overexpressing p53 in the fat body are not shown. According to the model, overexpressing p53 in the fat body should increase starvation sensitivity. To make the conclusions solid, this result should be provided. Alternatively, a reason for not conducting this experiment should be provided.

As suggested, we have now included data showing that conditional expression of Dmp53 in the FB of adult flies increased survival rates upon STV. We used the Gal4/Gal80ts system to avoid any deleterious effect of overexpression Dmp53 during development.

This new data has been included as Figure S6B and commented in text as follows:

Line 206: *“Finally, in accordance with a model where Dmp53 activation enhances survival, the conditional expression of Dmp53 in the FB of adult flies increased survival rates during nutrient deprivation (Fig. S6).”*

In panel E, the Lsp2>+ is not shown. Also the data is only shown for male flies. To make the conclusion of the myc-p53 interaction in starvation sensitivity convincing, the authors should provide the Lsp2>+ control, and the female data (even if negative). Or at least provide a reason why the female data was omitted.

As suggested, we have replaced Panel E by including the appropriate control flies. Additionally, we have now included data for both male flies (Fig. 6E) and female flies (Fig. S6).

Please indicate the fly gender also in the figure panels.

Modified

In line 182 is mentioned Fig 6F, but this panel is not included in figure 6.

Modified

A model figure explaining the dMyc-mir305-p53 pathway would be helpful for the reader.

A model integrating the mechanism regulating miR305 expression (Myc>pri-miR305) and processing (Foxo>Dicer1), and p53 activation in the fat body, according to nutrient availability has been included as Fig. 6F and explained as follows:

Line 258: “*In this scenario, dMyc promotes the transcription of pri-miR305, resulting in the repression of Dmp53 expression in the fat body of well-fed animals (Fig. 6F). Under nutrient deprivation, dMyc levels decrease, and FOXO becomes activated, impairing miR-305 expression and processing. This, in turn, leads to the activation of Dmp53, promoting energy homeostasis and enhancing animal survival under nutrient stress (Fig. 6F).”*

Figure Legend: “dMyc stimulates the transcription of pri-miR305, leading to the repression of Dmp53 expression in the fat body of well-fed animals. Under nutrient deprivation, dMyc levels decrease, and FOXO is activated, impairing miR-305 expression and processing. As a consequence, Dmp53 is activated, which facilitates energy homeostasis and supports animal survival during nutrient stress.”

**Reviewer #3:** In this MS, Gervé and cols analyze the contributions of the transcription factor Myc to the expression of miRNAs. They use Drosophila as a model to perform this analysis. They find that changes in Myc activity result in profound changes in miRNA expression. They also show that central elements controlling miRNA biogenesis are also altered upon changes in Myc activity. At the end of the MS, the authors focus their attention in miR-305 and its regulation by Myc in a context of starvation resistance.

The MS is, in general, and explores a mechanism of central interest in the context of animal development and homeostasis. However, several issues need to be addressed befor it is ready for publication.

We appreciate the reviewer's positive comments on our work and all the suggestions that have greatly improved the quality of the manuscript.

See specific comments below:

In the first part of the MS, the authors analyze the expression of a selected group of miRNAs in a context of Myc downregulation. However, the authors do not explain the criteria used to select those miRNAs. An explanation should be included.

Our bioinformatic analysis utilizing dMyc ChIP-Seq datasets revealed that approximately 60% of miRNA genes (142 out of 253) were found in close proximity to a dMyc peak region. To confirm this interaction and determine if the expression of these miRNAs was indeed regulated by Myc, it would have been valuable to conduct a small RNA transcriptome analysis. Unfortunately, due to budget constraints, this was not feasible for our study. As an alternative approach, we performed ChIP and qRT-PCR experiments using a limited, yet representative, number of miRNAs. The miRNAs were randomly chosen, and for most of them, we utilized oligos that were readily available in our laboratory.

We have clarify this in text as follows:

Line 92: “*We then measured the levels of primary miRNA (pri-miRNA) for a randomly selected group of miRNA genes by qRT-PCR and observed that expression of almost every tested pri-miRNAs was downregulated in GSG162>mycRNAi animals (Fig. 1B).*”

The authors analyse the changes in the expression in pre-miRNAs and pri-miRNAs. Although pri-miRNAs are explained in the introduction, pe-miRNAs are not introduced. To facilitate the understanding by non-specialists, a proper introduction of these terms (pri-, pre-, and mature miRNAs) will be required.

As suggested we have included a better description of miRNA biogenesis as follows:

Line 51: “*Primary miRNAs (pri-miRNAs) are transcribed by RNA Pol II and folded into a hairpin-like structure, facilitated by the presence of complementary sequences. The hairpin structure of the pri-miRNA is recognized and processed by an enzyme called Drosha, resulting in the production of a shorter RNA molecule called a precursor miRNA (pre-miRNA). The pre-miRNA is then exported from the nucleus to the cytoplasm, where it undergoes further processing by Dicer-1 (Dcr-1), leading to the production of ∼22-nt miRNA duplexes. Once loaded into the Argonaute-1 (AGO-1) containing miRNA-Induced Silencing Complex (miRiSC), the mature single-stranded miRNA guides the miRISC to the 3′ untranslated region (UTR) of target mRNAs [11–13].”*

Fig 1C, D, shows changes in a reduced number of miRNAs. Why only those? There is lack of cohesion between the results shown in B and C,D . I would suggest covering the analysis of pri-, pre-, and mature for the same set of miRNAs. This would provide a more complete view of this process.

As recommended, we have included additional data showing the levels of pre-miRNAs (Fig. 1D) for the same set of miRNAs analyzed in Figure 1B.

We have also included new data on Myc overexpression experiments to match the same number of pri-miRNAs analyzed in the Myc LOF experiments (new Figure S1B).

Previous studies by the Milán group showed that dMyc regulate bantam (PMID: 18451803) are consistent with this study. Those results should be mentioned here.

We have now properly cited this article and commented in text as follows:

Line 152: “It is noteworthy that a previous report indicated that dMyc overexpression in the wing primordium leads to an increase in the activity levels of bantam (29). We then extended our analysis to include alternative miR-GFP sensors based on ……”

The second miRNA shown is 219 (in C) and 279 (in D). Is that a typo or is it correct? The other 3 miRNAs shown are the same in both graphs.

We have included a new Fig. 1C solving this issue.

I would also suggest merging Fig 1 and Fig S1. The results shown are complementary and having them together will provide a more complete picture of the regulatory roles of dMyc.

We sincerely appreciate the suggestions made by the reviewers regarding the inclusion of additional data in Figure 1. However, we would like to highlight that only a small number of miRNAs show upregulation in Myc gain-of-function (GOF) experiments when compared to Myc loss-of-function (LOF) experiments. Incorporating this panel into Figure 1 would result in a crowded image, potentially making interpretation challenging. Nonetheless, if the reviewer strongly believes it is appropriate, we are more than willing to merge Figure 1 and Figure S1.

Fig 2 shows that Myc bind the promotor of numerous miRNAs. If this specific for miRNAs or a similar binding can be observed in other classes of genes?

We greatly appreciate the reviewer for pointing this out. In order to investigate the association between dMyc and miRNA genes, we conducted an enrichment analysis using the hypergeometric distribution. The results revealed a significant over-enrichment of miRNA genes, with a 2.64-fold enrichment compared to random expectations (hypergeometric p-value = 7.7e-25). These findings strongly support the highly significant association between dMyc and miRNA genes.

We have discussed this point in text as follows:

Line 119: *“An enrichment analysis based on the hypergeometric distribution revealed a significant over-enrichment of miRNA genes, with a fold enrichment of 2.64 compared to random expectations (hypergeometric p-value = 7.7e-25). This finding strongly supports the highly significant association between dMyc and miRNA genes.”*

Fig 3. Does Myc over-expression increase those transcripts? This complementary analysis might help to understand the results shown in Fig S1.

As suggested (also by reviewer 2), we have incorporated the measurement of ago-1, drosha, and dicer-1 mRNA levels in dMyc gain-of-function (GOF) experiments.

This data has been included in Fig. 3B and explained in text as follows:

Line 131: “*Interestingly, the transcript levels of dcr-1, drosha and ago1 were strongly reduced in larvae expressing mycRNAi (Fig. 3A), whereas they were upregulated in tub>dMyc animals (Fig. 3B).”*

Figure Legend: “*(A-B) qRT-PCR showing dcr1, drosha and ago1 mRNA levels in GSG162>mycRNAi larvae treated with RU486 or vehicle (A), as well as in tub>dMyc larvae (B). Results are expressed as fold induction relative to control animals. Three independent replicates were carried out for each sample.*”

In line 130, the authors mentioned: “The Drosophila ortholog of mammalian p53 (Dmp53) expression has previously been shown to be under control of miRNAs in both the wing imaginal disc and the fat body [28]. Interestingly, the levels of 3 miRNAs (miR-283, miR-219, miR-305) previously shown to regulate Dmp53 expression in the wing primordium [28] were reduced upon mycRNAi expression (Fig. 1B) and showed dMyc binding to its genomic region (Fig. 2D, Table S1).” This is followed by “We then used the larval wing primordium to characterize dMyc contribution in miRNA biogenesis.” I can´t find a logic connection between these two parts.

We have rephrased this sentence to make sense with the general idea as follows:

Line 144: “We then made use of a miRNA sensor that exploits the regulation of p53 expression by miRNAs in the larval wing primordium to characterize dMyc contribution in miRNA biogenesis.”

The authors showed that Myc controls miRNA expression and biogenesis. The results shown in Fig 4 bring them to conclude that Myc regulates the miRNA machinery. However, the changes shown in this Fig could partially result from changes in the expression of miRNAs and not only due to the biogenesis machinery. I would suggest the authors to consider that option.

Thank you for bringing this to our attention. We have revised the conclusion as follows:

Line 158: “*These findings indicate that the regulatory influence of dMyc extends beyond miRNAs targeting Dmp53. Moreover, our results indicate that dMyc plays a crucial role in regulating miRNA biogenesis in the Drosophila wing primordium, influencing either miRNA gene expression or processing.*”
